# Application of a new scan body for face‐driven fixed prosthetics

**DOI:** 10.1002/cre2.483

**Published:** 2021-08-25

**Authors:** Naruto Otawa, Tsukasa Aoki, Tomoki Sumida, Tsukasa Yanagi, Hirofumi Kido

**Affiliations:** ^1^ Medical Corporation Kohwakai OOTAWA Dental Clinic Maxillofacial Implant Center Fukuoka Japan; ^2^ Section of Oral Implantology, Department of Oral Rehabilitation Fukuoka Dental College Fukuoka Japan; ^3^ Department of Periodontology, Division of Oral Rehabilitation, Faculty of Dental Science Kyushu University Fukuoka Japan; ^4^ Section of Oral and Maxillofacial Surgery, Division of Maxillofacial and Diagnostic and Surgical Sciences, Faculty of Dental Science Kyushu University Fukuoka Japan

**Keywords:** CAD/CAM, dental implants, edentulous, oral rehabilitation

## Abstract

**Objective:**

The current method of digitally designing dental prostheses mainly focuses on intra‐oral soft and hard tissues, although the harmony of the facial soft tissue and the prosthesis is crucial, especially for esthetics. Here, we introduce a new method of digitally designing dental prostheses using a new device that generates a virtual patient and incorporates facial features into the prosthetic design.

**Materials and methods:**

A new extra‐oral scan body for facial scanning was designed and developed. A definitive edentulous maxilla implant cast with four extra‐oral scan bodies (regions: maxillary left and right lateral incisors, maxillary left and right premolars) was placed in the mouth of a dental mannequin. The dental mannequin was scanned with and without the extra‐oral scan bodies. For reference data, an impression of the maxilla was taken and scanned with a laboratory scanner. By superimposing each acquired data, a virtual patient was generated, and the spatial location of the abutments relative to the face was clarified. Identifying the accurate location of the abutments enabled to design face‐driven dental prosthesis.

**Results:**

Based on the color‐coded deviation map created by the data acquired from conventional and extra‐oral scan bodies, the divergence of the two data was mostly within 0.1 mm, which proves that the extra‐oral scan bodies were as accurate as conventional scan bodies. Therefore, the facial scan data and the scan data of the oral cavity were successfully superimposed, which allowed to generate a virtual patient to design face‐driven prosthesis.

**Conclusion:**

The new method is effective for designing high‐quality face‐driven prostheses, especially when treating a patient with a full‐arch implant‐fixed prosthesis.

## 1 INTRODUCTION

Advancements in digital technology in dentistry have resulted in innovations in dental treatment (Mangano et al., 2016), such as computer‐aided design and manufacturing (CAD/CAM). CAD/CAM has rendered the process of fabricating dental prostheses simple and less time‐consuming than conventional methods (Alshawaf et al., 2018; Lee et al., 2014; Van Noort, 2012; Yuzbasioglu et al., 2014). To initiate the CAD/CAM workflow, a digital impression is required. Technological advancements have introduced wide variations in the acquisition of digital impressions, ranging from intra‐oral scanners (IOS) to computed tomography (CT), depending on the purpose (Polido, 2010).

When designing dental prostheses, either digitally or conventionally, information regarding the intra‐oral soft and hard tissues serves as the main reference. However, when considering the esthetic aspects of the prosthesis, this information is insufficient. Analyzing and incorporating the patient's facial soft tissue is also essential for the esthetics of the prosthesis, as the harmony between the facial features and the dental prosthesis is crucial (De Smit & Dermaut, 1984; Esposito, 1980; Mack, 1996; Pound, 1951; Rosati et al., 2012). Photographs of the patient's face are frequently used as a reference for designing prostheses, but only limited information can be acquired given the two‐dimensional nature of the information.

The issue with the conventional workflow is that information related to the patient's facial soft tissue is missing. This would make it difficult to design and fabricate a highly esthetic prosthesis. Therefore, in order to design a highly functional and esthetic dental prosthesis, acquiring three‐dimensional (3D) information of the patient's facial anatomical features is crucial. To make full use of this 3D information, a digital approach can be used to examine the facial anatomical features from multiple angles. Some methods have already incorporated 3D data of a patient's facial anatomical features to generate a “virtual patient” for 3D clinical evaluation, as well as to design and fabricate dental prostheses (Hassan et al., 2017; Mack, 1996; Rosati et al., 2012). These are also very effective methods for generating virtual patient and to design prostheses, however, these methods did not consider facial soft tissue as an important factor. Therefore, we have focused on incorporating 3D facial soft tissue data of the patient into the CAD/CAM process. In order to incorporate digital 3D facial soft tissue data into the CAD/CAM process, a new scan body that can identify the spatial position of the implant abutment relative to the facial features for superimposing the digital wax‐up on the facial 3D data has been designed.

This article introduces a new device and method to generate a virtual patient and to incorporate the facial soft tissue for digitally designing implant‐fixed dental prostheses.

## 2 MATERIALS AND METHODS

Since the evaluations of the extra‐oral scan body were conducted using a mannequin and no human subjects or animals were involved, ethical approval and informed consent was not required. This decision by the authors were based on Ethical Guidelines for Medical and Health Research Involving Human Subjects, created by the Ministry of Education, Culture, Sports, Science and Technology and the Ministry of Health, Labor and Welfare of Japan.

The guideline used to plan, conduct and report was SQUIRE.

### 2.1 Constructing the workflow for the new digital design system

To understand the new digital workflow system using the extra‐oral scan body, it is necessary to understand the conventional CAD/CAM workflow for implant‐fixed prostheses. The conventional workflow begins by scanning the dental stone cast model with attached conventional scan bodies using a laboratory scanner or by directly scanning the patient's intra‐oral tissue with conventional scan bodies attached using an IOS. The scanned data are then converted into a standard tessellation language (STL) file format to be used in CAD software. After, the design of the dental prosthesis is altered and confirmed, the CAD data are converted into an STL file format and sent to the milling machine to fabricate the dental prosthesis (Joda & Brägger, 2014).

**Figure 1:**
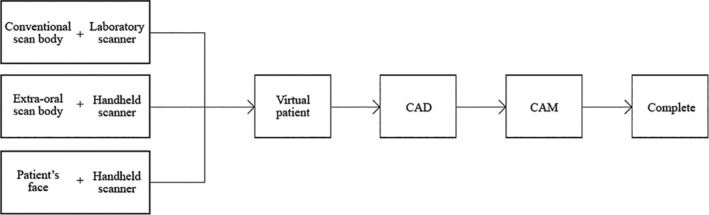
Workflow chart of the new system. By utilizing three scan datasets, a virtual patient is generated, and the prosthesis is designed. Once the design is confirmed, the prosthesis is manufactured. Most steps are performed digitally. CAD, computer‐aided design. CAM, computer‐aided manufacturing

The majority of the steps in the new system are the same as those in the conventional system, with some exceptions. The steps are as follows:

1. The patient's face is scanned with the handheld scanner while ensuring that the patient is displaying a broad smile for the initial scanning. The data will be called “Data A.”
2. The extra‐oral scan body is attached and another digital impression of the patient's face is made in order to identify the spatial location of the abutment (“Data B").
3. Information on the location of the abutment and intra‐oral hard and soft tissues will be obtained by scanning the stone cast using a desktop scanner (“Data C").
4. By superimposing Data A and B, the spatial location of the abutment relative to the face is clarified (“Data D").
5. By superimposing Data B and C, the location of the abutment within the oral cavity will be clarified (“Data E"). With Data D and E superimposed based on the abutments, the digital wax‐up of the prosthesis designed with CAD software can be accurately placed inside the virtual patient's mouth. This new system is available for any patient treated with dental implants. The steps and the simplified flow chart are shown in Figure 1.

By utilizing these data and following the steps above, a virtual patient is generated, allowing the clinician and dental technician to simulate and design the dental prosthesis using data from the intra‐oral soft and hard tissues and facial soft tissue to evaluate how the dental prosthesis affects the facial features.

### 2.2 Designing and fabricating the extra‐oral scan body

**Figure 2:**
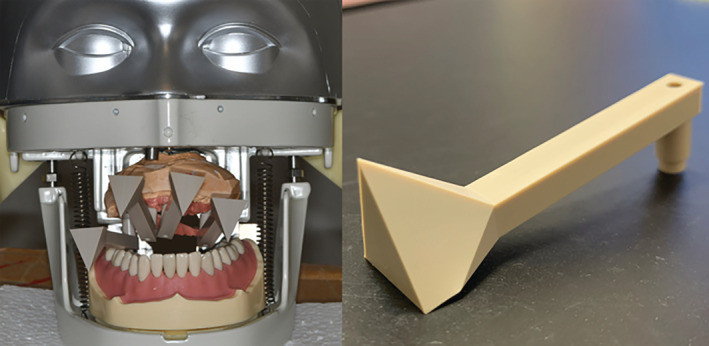
(Right) Whole view of the extra‐oral scan body. Polyetheretherketone was used to fabricate this scan body. (Left) Front view of the dental mannequin. Definitive cast with extra‐oral scan bodies attached is set in the mouth

**Figure 3:**
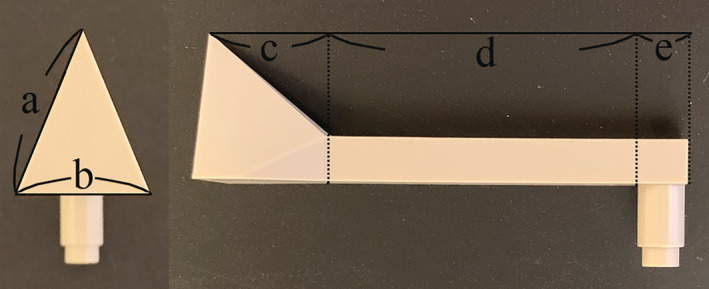
Parts of the extra‐oral scan body. (a) Legs (extra‐oral triangle), (b) base of extra‐oral triangle, (c) extra‐oral region, (d) pillar region, (e) base region

Simple geometrical shapes were selected when designing the new device to enable the handheld 3D optical scanner to scan the structure of the extra‐oral scan body, thereby decreasing the probability of acquiring distorted digital data (Salvi et al., 2010). The structure of the extra‐oral scan body consists of three components: the extra‐oral scan region, pillar region, and base region (Figures 2 and 3). The specific dimensions of the extra‐oral scan body and the names of the parts are listed in Table 1 and Figure 3.

**Table 1:** Specific dimensions of the extra‐oral scan body

	Legs (extra‐oral triangle) (mm)	Base (extra‐oral triangle) (mm)	Extra‐oral region (mm)	Pillar region (mm)	Base region (mm)
Extra‐oral scan body	20	15	15	40	7

Polyetheretherketone (PEEK) was selected for manufacturing the extra‐oral scan body given its physical and mechanical properties, such as excellent flexural fatigue resistance and sufficient stiffness for the structural rigidity of the structure (Najeeb et al., 2016; Rae et al., 2007). Moreover, PEEK is more accurate than other dental materials for 3D scanning (Arcuri et al., 2020). The extra‐oral scan bodies were manufactured with the help of Brain Base Corporation (Tokyo, Japan).

### 2.3 Verification of scan data accuracy

We created a definitive cast of an edentulous maxilla based on an edentulous model. Four extra‐oral scan bodies of the same size were attached to the cast. The implants were in the maxillary left and right lateral incisors, and maxillary left and right second premolars. The dental stone cast with extra‐oral scan bodies attached was then attached to a dental mannequin (Figure 2). The 3D scan data of the mannequin with the stone cast were obtained using a handheld 3D scanner (EinScan Pro‐plus, Shining 3D; Hangzhou, China). Before the scan was conducted, the handheld scanner was calibrated to maximize its accuracy.

**Figure 4:**
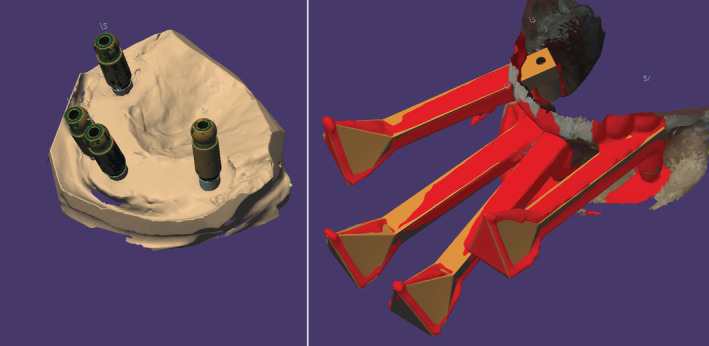
(Left) Three‐dimensional (3D) control data acquired using a laboratory scanner and conventional scan bodies. Green represents the data acquired by a laboratory scanner. Beige represents computer‐aided design (CAD) data. (Right) 3D test data acquired using a handheld scanner and extra‐oral scan bodies. Red represents the scan data acquired by a handheld scanner, and beige represents CAD data

**Figure 5:**
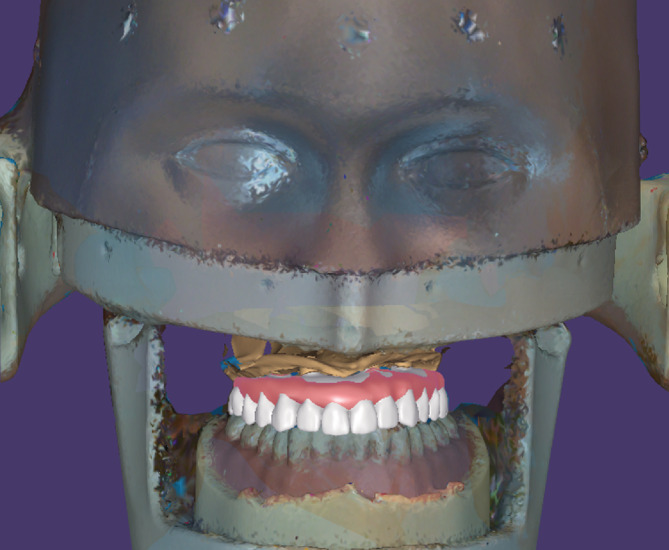
A virtual patient is generated based on the acquired scan data. The CAD data of the prosthesis are successfully superimposed based on the abutment

Data on intra‐oral soft and hard tissues were acquired by scanning the dental stone cast with the attached conventional scan bodies (DESS; Granite Bay, CA) using a laboratory scanner (PRIME, DOF; Seoul, Korea). The scan data acquired by the laboratory and handheld scanners were termed “reference data” and “scan data,” respectively. The process of identifying the location of the dental abutment is shown in Figure 4. After obtaining the data on abutment location, a digital wax‐up was superimposed on the virtual patient's face (Figure 5).

## 3 DISCUSSION

**Figure 6:**
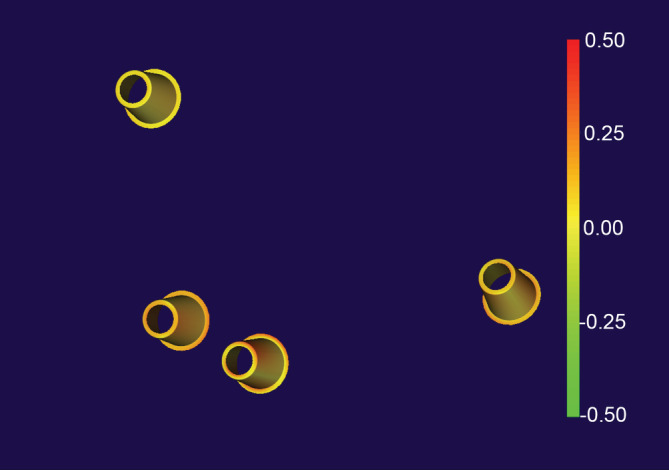
Color‐coded deviation maps made by superimposing reference data and scan data based on the abutment level using a best‐fit algorithm. Green indicates inward displacement, red shows the outward position of the mesh compared to the reference data, and yellow shows the absence of change. Divergence in the two datasets fell within 0.1 mm

In this new system of designing and fabricating implant‐fixed dental prostheses, the dental implant abutment is used as a landmark to superimpose the digital wax‐up of the prosthesis and 3D facial data. The abutment was used because the CAD software was unable to export the STL file format of the implant body. However, the CAD implant abutment data were exportable in the STL file format; thus, the abutment was selected as a landmark for superimposition. Although a virtual patient can be generated, the accuracy of extra‐oral scan bodies should be high in order to be useful. To verify the accuracy of the new device, a color‐coded deviation map of the superimposed abutments was created by using the data acquired from conventional and facial scan bodies (Figure 6). Most of the color in the map was yellow, indicating that the divergence of the two data was mostly within 0.1 mm, suggesting good accuracy of the device.

The mannequin used in this trial may not have been sufficient to prove that the new device and the facial features enable the design of highly esthetic dental prostheses since it lacks several main extra‐oral features. However, as it could generate a virtual patient and design a full‐arch implant‐fixed prosthesis from the limited extra‐oral information, it has great potential in creating a highly esthetic dental prosthesis. Further confirmation is needed with future studies.

### 3.1 Limitations of this technique

Although the scan body is important for identifying the dental implant location, the scanner employed to acquire digital 3D information of facial soft tissue is another important factor that influences accuracy (Eiríksson et al., 2016; Geng, 2011; Georgopoulos et al., 2010; Modabber et al., 2016). In this study, we used one type of handheld 3D scanner, but the outcomes may have differed if different handheld scanners were used given the distinct camera features of each scanner (Amornvit & Sanohkan, 2019). Generally, there are two types of 3D scanners: laser scanners and structured light scanners. The scanner used in this study was a structured light scanner, which projects light patterns on the subject to determine the 3D structure of the target. This scanner features fast scan times, a large scanning area, high accuracy, and is safe to use around the eyes. From the acquired results, it is highly recommended to use a structured light scanner that has the same or higher specificity than the one used in this study in order to gain accurate results.

The proficiency of the operator is also an important factor. IOSs are not affected by the operator (Arcuri et al., 2020); however, from past studies, it is known that the proficiency of the operator affects data accuracy for extra‐oral scanning. Therefore, the operator needs to be highly proficient in order to gain the 3D information necessary to generate a virtual patient. It is highly recommended to practice using the handheld 3D scanner before clinical use.

In this study, a mannequin was used to confirm that the new device could be used to create a virtual patient for planning and designing prostheses. However, when utilizing this new device in clinical situations, one should consider that, unlike the mannequin, an actual patient could make slight movements during acquisition of facial data, which would result in motion artifacts. The motion artifacts could be a challenge in actual clinical practice. Though we have not yet determined how motion artifacts will affect the data, it could be expected that it would cause adverse effects. For example, slight change of the position of the abutment might occur compared to the actual position of the abutment. Therefore, in order to avoid consequences caused by motion artifacts, such as redesigning and remanufacturing of the final prosthesis, it is highly recommended that conducting a try‐in based on the acquired data should be done first to confirm that the design is functionally and esthetically fine before moving on to final processing. In order to clarify the effect of motion artifacts on the acquired data, further confirmation should be conducted with actual patients.

### 3.2 Clinical relevance

Reports on IOS accuracy indicate that deviations in the acquired data become more distinctive with a certain distance between implants (Amin et al., 2017; Flügge et al., 2016; Giménez et al., 2015; Miyoshi et al., 2020; Vandeweghe et al., 2017). With an extra‐oral scan using a handheld 3D optical scanner, consideration of the error caused by the distance between implants during digital data acquisition is not required, suggesting that extra‐oral scanning is a superior choice for treatment planning in patients with a full‐arch implant‐fixed prosthesis. The new workflow requires the use of a laboratory scanner to scan the definitive implant cast attached to the conventional scan body. Scan bodies on lab analogs exhibit higher reproducibility of fit than original implants, leading to more accurate data (Stimmelmayr et al., 2012). This suggests that, when acquiring intra‐oral data from patients, it is better to obtain data from the definitive cast rather than from a direct scan of the patient's oral cavity. Therefore, this new method can potentially demonstrate higher accuracy when treating patients with a full‐arch implant‐fixed prosthesis compared to using an IOS.

The accuracy of the virtual patient affects treatment outcomes because the dental prosthesis is fabricated based on CAD data. Several methods of generating virtual patients have been introduced and reported in past studies. According to Joda et al. (2015), three different sets of 3D data are needed to create a virtual patient: the facial skeleton, extra‐oral soft tissue, and dentition including intra‐oral soft tissue. At least two of these three datasets are necessary to generate a virtual model of the patient. The extra‐oral tissue and facial skeleton were the two most common datasets used to generate virtual patients, requiring the use of CT. In this new method of designing a full‐arch implant‐fixed prosthesis using an extra‐oral scan body, the datasets selected were extra‐oral and intra‐oral soft tissue and dentition because extra‐oral scanning is non‐invasive, repeatable, and the virtual patient generated using these data facilitates the prediction of treatment outcomes.

## 4 CONCLUSIONS

This article describes a new method of generating a virtual patient using a new device. This newly designed extra‐oral scan body could enable clinicians to design and fabricate face‐driven and highly esthetic dental prostheses using digital technology. The new digital workflow is low‐cost and effective for designing high‐quality face‐driven prosthetics, especially when designing and fabricating full‐arch fixed prostheses.

## CONFLICT OF INTEREST

The authors declare that they have no conflict of interest.

## AUTHOR CONTRIBUTIONS

Naruto Otawa contributed to conception, design, and critically revised the manuscript; Tsukasa Aoki contributed to the acquisition and analysis of the data and led the writing; Tomoki Sumida contributed to acquisition, analysis of the data, and critically revised the manuscript; Tsukasa Yanagi contributed to the acquisition of the data; Hirofumi Kido contributed to conception, summarizing and analyzing the data, and critically revised the manuscript.

## Data Availability

The data that support the findings of this study are available from the corresponding author upon reasonable request.
